# Duplex PCR assay for the detection of avian adeno virus and chicken anemia virus prevalent in Pakistan

**DOI:** 10.1186/1743-422X-8-440

**Published:** 2011-09-19

**Authors:** Latif U Rehman, Bakht Sultan, Ijaz Ali, Muhammad A Bhatti, Sana U Khan, Khaliq U Zaman, Anila T Jahangiri, Najib U Khan, Aqib Iqbal, Jehan Bakht, Zahoor A Swati, Muti U Rehman

**Affiliations:** 1 Institute of Biotechnology and Genetic Engineering, KPK Agricultural University, Peshawar, 25000, Pakistan; 2 GP (Grand Parent) Laboratory, GOR5, Lahore, 54770, Pakistan; 3 Kohat University of Science and Technology, Kohat, 26000, Pakistan; 4 University of Veterinary and Animal Sciences, Lahore, 54000, Pakistan

## Abstract

Avian Adeno viruses and Chicken Anemia Viruses cause serious economic losses to the poultry industry of Pakistan each year. Timely and efficient diagnosis of the viruses is needed in order to practice prevention and control strategies. In the first part of this study, we investigated broilers, breeder and Layer stocks for morbidity and mortality rates due to AAV and CAV infections and any co-infections by examining signs and symptoms typical of their infestation or post mortem examination. In the second part of the study, we developed a duplex PCR assay for the detection of AAV and CAV which is capable to simultaneously detect both the viral types prevalent in Pakistan with high sensitivity and 100% specificity.

## Introduction

Adenoviruses are common infectious agents of poultry. The avian adenoviruses are divided into three groups i.e. group Ι, group Π and group III. Group Ι is composed of 5 species (A to E) and 12 serotypes (FAdV-1 to FAdV-12) of avian adenoviruses of chickens, turkeys, goose and duck [1-3]. The group Ι adenoviruses are famous for causing hydropericardium-syndrome in chickens (caused by FAdV-4 strains), Quail Bronchitis in quail (caused by FAdV-1) and inclusion body hepatitis [4-6]. Group Π of avian adenoviruses causes hemorrhagic enteritis in turkeys, marble spleen disease in pheasants and splenomegaly in chickens. Group Π viruses have a common group antigen that differentiates them from group Ι [7]. Group III viruses are famous for egg drop syndrome in chickens and a similar virus is believed to infect duck [8].

The first avian adenovirus was isolated in 1949 when material from a case of lumpy skin disease in cattle was inoculated into embryonated chicken eggs [9]. Other early unintentional isolates of fowl adenoviruses were the chicken embryo lethal orphan (CELO) isolates made in embryonated eggs [10] and the GAL viruses from chicken cell cultures [11]. The first isolate of an avian adenovirus from diseased birds was from an outbreak of respiratory disease in bobwhite quail (*Colinus virginianus*) by Olson [12].

The avian adenovirus infections cause high economic losses by increasing mortality in chickens, poor feed conversion, drop in egg production, eggs of poor quality and also diminished weight gain. The avian adenoviruses are also involved in immune suppression which may lead to secondary infections [13-15].

Chicken anaemia virus, previously known as chicken anaemia agent (CAA), was first isolated by Yuasa et al in 1979 [16]. The virus is small, non-enveloped, icosahedral, and contains a circular, single-stranded DNA genome [17,18]. It has recently been classified as the sole member of the genus *Gyrovirus *[19]. It causes severe aplastic anaemia in young chickens [16,20-22], depletion of lymphoid organs, subcutaneous and intramuscular haemorrhages, and destruction of erythroblastoid cells in bone marrow [23-25]. In addition to the above, some specific symptoms are also observed like haemorrhages in leg and chest muscles, focal necrosis in liver, ulcerative erosions in gizzard, and necrosis of wing skin [26].

Both AV and CAV have been implicated to cause serious economic losses to the poultry Industry of Pakistan [27]. Adenoviruses and Chicken Anaemia viruses are diagnosed in a number of ways including Electron microscopy, Insitu hybridization, Virus neutralization, ELISA, Immunofluorescence and Immunoperoxidase tests [28-33]. Majority of the existing methods used for the detection of adenovirus and chicken anaemia viruses are either highly expensive, time consuming or less reliable for the qualitative detection of different viral genotypes prevalent in Pakistan. Due to limitations of the Multiplex approach [34] for the efficient diagnosis of the viral genotypes prevalent in Pakistan, we conducted this study in order to develop an efficient, less time consuming and cost effective duplex PCR assay for the detection of adenovirus and chicken anaemia viruses in poultry birds which is capable to detect the prevalent viral types in Pakistan. Moreover, we also investigated broiler, breeders and Layer birds from various poultry farms of the country for the presence of AAV or CAV infection.

## Materials and Methods

### Field Samples and Experimental birds

The current study was carried out at GP laboratories Lahore after having approved by the Board of study of the Institute of Biotechnology and Genetic Engineering, Peshawar. The study was conducted in accordance with internationally accepted guidelines. Experimental birds and field samples were provided by various poultry farms from across the country. Chicks were artificially infected with various genotypes of AV and CAV and slaughtered after the signs and symptoms of the disease appeared. Liver, thymus and bone marrow samples were collected from diseased chickens at Grand Parents Laboratory (GP Lab) Lahore, where chickens and other birds are brought for the diagnosis of various diseases from different commercial farms from all over the country. Apparently healthy and effected Broiler, Layer and breeder stocks in various poultry farms across the country were also investigated for the presence of AAV and CAV infections by appearance of the clinical signs and symptoms of the diseases or post mortem examination. The morbidity and mortality rates in the case of AAV and CAV were also recorded. Determination of any co-infection was carried out microbiologically using standard procedures.

### DNA extraction

Adenovirus DNA was extracted from liver and Chicken Anemia Virus DNA from thymus and bone marrow samples using QIAamp^R ^DNA Mini Kit (Qiagen GmbH, D-40724, Hilden, Germany) according to the manufacturer's instruction. The DNA was then stored at -20°C until used.

### Oligonucleotide Primers

Two sets of primers H1 (5'-TGGACATGGGGGCGACCTA-3') and H2 (5'-AAGGGATTGACGTTGTCCA3') for AAV, MK10 (5'-GACTGTAAGATGGCAAGACGAGCTC-3') and MK11 (5'-GGCTGAAGGATCCCTCATTC-3') for CAV [35,34] were synthesized by Invitrogen (Carlsbad, California) which were used to amplify 1219 bp and 675 bp from their respective viral genomes.

### PCR amplification

Duplex PCR was conducted in a 25 μl reaction volume, containing 12.5 μl 2 × PCR Master Mix (Fermentas, Canada), 1 μl (10 μM) each of the Primer (H1, H2, MK10 and MK11), 1 μl each template DNA (from both AAV and CAV) and 6.5 μl nuclease free water.

PCR was performed in an automatic DNA thermal cycler (Techne^® ^Endurance TC-312). The cycling protocol consisted of an initial denaturation at 95°C for 5 minutes followed by 35 cycles at 95°C for 45 sec, 55°C for 45 sec and 72°C for 1.5 min. Final extension was carried out at 72°C for 10 min. Positive and Negative controls were included each time the amplification was carried out.

### Detection of PCR products

PCR products were detected on 1% agarose gel pre-stained with Ethidium Bromide at 110 V for 20 min. 100 bp ladder (Gibco BRL) was used as a DNA size marker.

### Specificity and Sensitivity of the duplex assay

Specificity of the duplex PCR was assessed by examining its ability to amplify only AV and CAV genomes. Each duplex assay performed included two simplex reactions in which AAV primers were used to amplify the CAV DNA while the CAV primers were mixed with AAV DNA in another reaction tube to check if there was any non-specific amplification. The AAV and CAV-specific primers were also used to amplify other viral and bacterial samples available at GP Lab. The sensitivity of the duplex assay was investigated by serially diluting viral DNA (10 ng, 8 ng, 6 ng, 4 ng, 2 ng .04 ng, .03 ng) stock of both AAV and CAV DNA.

## Results

In current study we observed avian adenoviral infection in broilers and breeders while no case of avian adenovirus in layers was observed. The mortality due to AAV varied in various broiler farms from 3% to 90%. In contrast we observed no incidence of CAV in breeders while in case of broiler we found CAV in stunted birds having no morbidity and significant mortality. In layers (6-8 weeks) we observed up to 30% mortality. The mortality was enhanced by secondary bacterial infection especially E. coli and Staphylococcus.

Results of the duplex PCR assay in the case of both the experimental birds and field samples indicated that the assay is highly specific for the detection of both AAV and CAV. A total of 300 samples each, taken from the experimental birds, in the case of AAV and CAV, were used for DNA extraction and subsequently subjected to the duplex assay. The specificity of the duplex assay was 100% for the detection of both the viruses (Table 1, 2). Field samples collected from poultry farms across the country were also used for the detection of AAV or CAV using the duplex assay. All the birds samples used for DNA extraction had clinical manifestations of AAV or CAV infections. A total of 100 field samples in the case of AAV revealed that 96 out of 100 were positive for AAV DNA by duplex PCR. Only 4 samples which showed the signs and symptoms typical of AAV infection turned out to be negative by the duplex PCR.

**Table 1:** Adenoviral samples tested by duplex PCR

Source of Samples	No. of samples	PCR positive	Organ used for DNA Isolation
Field samples	100	96 (96%)	Liver

Experimental samples	300	300 (100%)	Liver

Total	400	396 (96%)	

**Table 2:** CAV (Chicken Anemia Virus) samples tested by duplex PCR

Source of Samples	No. of samples	PCR positive	Clinical Manifestation/Organ (Used for DNA Isolation)
Field samples	100	95 (95%)	+/bone marrow, thymus, spleen

Experimental samples	300	300 (100%)	+/bone marrow, thymus, spleen

Total	400	395 (95%)	

Almost similar results were obtained after the clinically validated organ/Tissue samples were investigated for CAV DNA by the duplex assay. All the 300 experimental samples were positive for CAV DNA by the duplex assay while out of the 100 field samples, 95 turned out to have active CAV infection (Table 2).

Specificity of the duplex PCR was determined by examining its ability to detect and differentiate only AV and CAV. Each time the duplex assay was carried out, two simplex reactions were performed in such a way that AAV primers were used to amplify the CAV DNA and the CAV primers were mixed with AAV DNA in another tube to check if there appeared any amplified product. Apart from this, the primers were used to amplify other viral and bacterial samples available at GP Lab. In all the cases, we found out that the respective AAV and CAV primers were highly specific (100%) by amplifying only their respective genomes.

**Figure 1:**
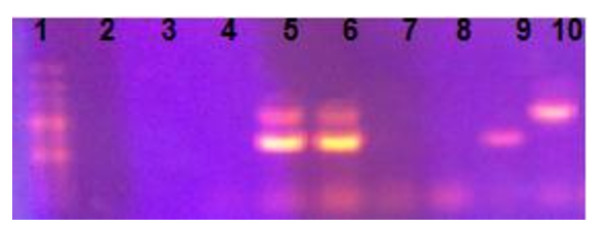
**Gel photograph of duplex PCR**. Lane 1 contains 100 bp Plus DNA ladder, Lane 2 contains AAV DNA with CAV primers, Lane 3 contains CAV DNA with AAV primers, Lane 4 is negative field sample, Lanes 5 & 6 are positive field samples for AAV (1219 bp) and CAV (675 bp), Lane 7 & 8 are negative controls, Lane 9 is CAV positive control while Lane 10 is AAV positive control.

To know about the sensitivity of both primer sets (H1-H2 and MK10-MK11) and its affinity for their target DNA, PCRs using each primer set were applied to the serially diluted DNAs of avian adenovirus and chicken anemia virus. Results of the duplex assay indicated that, 30 pg of AAV DNA and 40 pg of the CAV DNA was successfully amplified (Figure 1).

## Discussion

Avian adenovirus and chicken anemia virus cause heavy economic losses to the poultry industry in Pakistan. The mortality due to avian adenovirus has been reported up to 80% [36] while chicken anemia virus cause fewer deaths but dual infection are however very serious when the chickens are infected with other virus like IBD, Merek's disease, reovirus and adenovirus in addition to chicken anemia virus [37,38].

In this study we observed that Layer stocks at various poultry farms were free of AAV infection while the breeder and broiler stocks were infected showing 3-90% mortality in various poultry farms causing heavy economic losses on the farmers across the country. Investigation of the flocks for CAV infection indicated that the breeder stocks were free of the infection broiler birds were found to be infected. Interestingly, no morbidity was recorded but mortality was significantly high. In young Layers, mortality was recorded to be 30%. We also observed in this study that co-infections of birds with E.Coli and Staphylococci enhanced the rates of mortality among various flocks of birds.

Serological tests for the diagnosis of AAV and CAV like Indirect Immunoflourescent Assay, Enzyme-linked Immunosorbant Assay, Dot ELISA and Double Immuno-diffusion are difficult to be interpreted because antibodies against these viruses are found in both healthy and infected birds. These tests are also highly laborious, time consuming and also having low sensitivity and specificity [39] According to previous data available the sensitivity and specificity of the dot ELISA for the detection of avian adenovirus was 67.1% and 96.9% [40]. Similarly Indirect Immunofluorescence (IIFA) that takes only few hours is also reported to have poor sensitivity and specificity. Antibody-based tests are not informative about active infection [41] and therefore PCR is used to detect nucleic acids of the pathogens in order to confirm the status of infection.

In Pakistan, due to scarcity of technological expertise in modern diagnostics, avian infections are diagnosed either clinically or microbiologically; which very often leads to faulty or untimely diagnosis of deadly infections making the management of birds not only uneconomical but also sometimes threatening for the public health. Due to the economic importance of AAV and CAV for the poultry industry, we attempted in this study to develop a cost-effective duplex assay for the diagnostic set ups in our country and elsewhere. Serial dilutions of the AAV and CAV DNA extracted from the effected organs indicated that as low as 30 pg DNA could be efficiently detected by the duplex assay in the case of AAV while 40 pg of CAV DNA was enough for amplification of the 672 bp product. Specificity of the duplex assay was assessed by using the primers specific for AAV and CAV to amplify other viral and bacterial DNA isolated from tissues of birds at GP Lab. We observed that the primers used did not amplify non-specifically in any of the cases. The sensitivity and specificity of the assay developed is high enough to be used for screening stocks of birds for CAV and AAV infections in Pakistan. The duplex assay could also be used as a confirmatory test for the determination of active AAV or CAV infection in effected birds.

## Conclusion

Avian Adeno Virus and Chicken Anemia Virus infections cause high morbidity and morbidity in poultry birds across Pakistan which ultimately transpires in the form of heavy economic losses. Duplex PCR assay developed in this study has 100% specificity and high sensitivity to detect AAV and CAV infections in poultry birds and is a major improvement in poultry diagnostics of Pakistan in terms of its reliability and efficiency to detect viral types. It will not only save time to carry out timely diagnosis of the infections in Pakistan but will also save precious foreign exchange spent on confirmation of diagnosis from other countries.

## Competing interests

The authors declare that they have no competing interests.

## Authors' contributions

IA, BA and MAB designed the study and advised about the protocols. LR carried out sampling and experimental procedures. ATJ, AI, SUK, KUZ, MUR and NUK helped with experimental procedures and manuscript preparation. JB and ZAS critically reviewed the manuscript. All authors read and approved the final manuscript.
